# A cross-sectional nationwide survey of guideline based syncope units in the Netherlands: the SU-19 score—a novel validation for best practices

**DOI:** 10.1093/europace/euae002

**Published:** 2024-01-08

**Authors:** Steven van Zanten, Jelle S Y de Jong, Mike G Scheffer, Evert C A Kaal, Joris R de Groot, Frederik J de Lange

**Affiliations:** Department of Cardiology, Reinier de Graaf Gasthuis, Reinier de Graafweg 5, 2625 AD Delft, The Netherlands; Department of Clinical and Experimental Cardiology, Amsterdam Cardiovascular Sciences, Amsterdam University Medical Centre, University of Amsterdam, Heart Centre, Amsterdam, The Netherlands; Department of Cardiology, Reinier de Graaf Gasthuis, Reinier de Graafweg 5, 2625 AD Delft, The Netherlands; Department of Neurology, Maasstad Hospital, Rotterdam, The Netherlands; Department of Clinical and Experimental Cardiology, Amsterdam Cardiovascular Sciences, Amsterdam University Medical Centre, University of Amsterdam, Heart Centre, Amsterdam, The Netherlands; Department of Clinical and Experimental Cardiology, Amsterdam Cardiovascular Sciences, Amsterdam University Medical Centre, University of Amsterdam, Heart Centre, Amsterdam, The Netherlands

**Keywords:** Syncope units, Network, SU-19 score, Guideline implementation, Autonomic function tests, Head-up tilt test

## Abstract

**Aims:**

We aimed to identify all syncope units (SUs) in the Netherlands and assess the extent to which these SUs fulfil the essential requirements outlined by the consensus statements of the European Heart Rhythm Association and the European Society of Cardiology syncope guidelines. For this, we developed the SU-19 score, a novel guideline based validation tool for best practice.

**Methods and results:**

All outpatient clinics of cardiology, neurology, and internal medicine in the Netherlands were screened for presence of any form of structured specialized syncope care. If present, these were included as SUs and requested to complete a questionnaire regarding syncope care. We assessed all SUs using the SU-19 score regarding structure (3 points), available tests (12 points), and initial evaluation (4 points). Twenty SUs were identified in the Netherlands, both academic (5/20) and non-academic hospitals (15/20), 17/20 reported multidisciplinary involvement during initial evaluation. In 19/20, neurology, cardiology, or both were responsible for the syncope management. Non-physicians were involved performing the head-up tilt test (44%) and initial evaluation (40%). The mean SU-19 score was 18.0 ± 1.1, 45% achieved the maximum score of 19 points. Variations were observed in protocols for active standing test, carotid sinus massage, and head-up tilt test.

**Conclusion:**

There is a network of 20 SUs in the Netherlands. Forty-five per cent fully met the SU-19 score (mean 18.0 ± 1.1). Slight variety existed in protocols for autonomic function tests. Neurology and cardiology were mostly involved in syncope management. Non-physicians play an important role in syncope care.

## Graphical Abstract

**Graphical Abstract:**
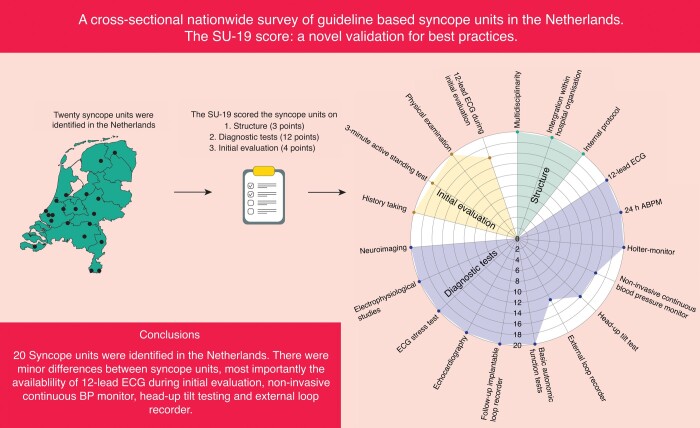

**What’s new?**

- The implementation of the European Society of Cardiology Guideline on syncope is feasible in both academic and non-academic outpatient syncope units.
- We developed a novel and feasible syncope unit-19 score to assess implementation of the European Heart Rhythm Association consensus statements for syncope units and European Society of Cardiology guideline on syncope.
- Both cardiology and neurology are in the lead for syncope care in the Netherlands.
- The role for non-physicians becomes more important and prevalent in staffing the syncope unit.

## Introduction

Syncope is a transient loss of consciousness (T-LOC) caused by hypoperfusion of the brain and has a plethora of different causes. The European Society of Cardiology (ESC) guidelines recommend the initial syncope evaluation for every syncope patient as the standardized approach.^1^ The initial syncope evaluation consist of complete, thorough history taking, physical examination including 3 min active standing test (AST), and a 12-lead electrocardiogram (ECG).^2^ Twenty years ago, the first European syncope guidelines provided a standardized approach for the evaluation of patients with syncope.^3^ A standardized structural approach results in a higher diagnostic yield and lowers healthcare costs by reducing additional tests and hospital admissions.^4–7^ However, implementation in routine medical care has proven complex. Most barriers appeared on the level of healthcare professionals and organizational context.^8^ Specialized syncope units (SUs) may be of help in providing the standardized assessment of syncope patients.^5^ A standardized approach to patients experiencing syncope is crucial as starting point on which treatment is based; however, how to handle the syncope patient is equally, if not more, important in order to prevent syncope recurrence. This involves considerations such as education to the patient, physiotherapy, physical counter-pressure manoeuvres, pharmacological treatment, pacemaker therapy and (possibly with the preferred algorithms and access to investigational methods such as cardioneuroablation).^1,9–22^

A consensus statement^2^ on SUs published by the European Heart Rhythm Association (EHRA) describes a SU as a facility featuring the following four items: (1) a standardized approach to the diagnosis and management of T-LOC and related symptoms, (2) dedicated staff, (3) access to appropriate diagnostics and therapies, and (4) the SU should take the lead in training and educating.^2^ We aimed to identify the current network of all SUs in the Netherlands and assess their implementation of guideline based syncope care. We emphasized on the first three items and did not include the aspect of training and educating (fourth item). It is also important to note that our focus did not extend to the treatment of patients with syncope. We analysed to which extent the units fulfil requirements as indicated by the EHRA consensus statements one and three and the current ESC syncope guidelines for the (initial-) syncope evaluation as the standardized approach.^1,2,19^

## Methods

This is a cross-sectional nationwide qualitative study in the Netherlands. We aimed to include all outpatient clinics in the Dutch hospitals with any form of dedicated care for patients with syncope/T-LOC. Therefore, we obtained a list of all (general and university) hospitals in the Netherlands from the national institute for public health.^8^ We then contacted all neurology, internal medicine, and cardiology outpatient clinics in all Dutch hospitals by phone and obtained information regarding specialized care for syncope patients.^23^ Hospitals with multiple locations were regarded as one hospital. All outpatient clinics that reported to have any form of dedicated care for syncope patients were included as SU in this survey and were requested to fill out a detailed questionnaire (see next section for details). Syncope units located in university hospitals were labelled as academic SU, all other hospitals were labelled as non-academic SU. The Dutch Autonomic Society (Werkgroep Syncope en Autonome Aandoeningen) was consulted to verify all the included SUs that were obtained in this survey. Multidisciplinary assessment was defined as: more than one specialty involved during the initial assessment.

### SU-19 score list for syncope units

We created a questionnaire consisting of 25 questions (see Supplementary material online, Supplementary*S1*) regarding syncope care. These include aspects of consensus statements one and three of the EHRA position paper^2^ and the ESC syncope guidelines.^1^ Consensus statement one is a comprehensive list regarding the structure and the availability of diagnostic tests. Consensus statement three contains requirements for the initial evaluation (see *Table 1*).

**Table 1:** List of the SU-19 score

Content of the SU-19 score (19 points)
Structure (3 points)
Multidisciplinarity
Integration within hospital organization
Internal protocol
Available diagnostic tests (12 points)
12-Lead ECG
24 h ambulatory blood pressure monitoring
Holter monitor
Non-invasive continuous blood pressure
Head-up tilt test
External loop recorder
Basic autonomic function test^a^
Follow-up implantable loop recorder
Echocardiography
ECG stress test
Electrophysiological studies
Neuroimaging
Initial evaluation (4 points)
History taking
3-min active standing test
Physical examination
12-Lead ECG

The items are derived from the European Heart Rhythm Association consensus statements one and three, and the European Society of Cardiology guidelines on syncope. Basic autonomic function tests consist of: Valsalva manoeuvre, deep breathing 30:15 ratio, the cold pressure, sustained hand grip, and mental arithmetic. Each item corresponds with one point.

ECG, electrocardiogram.

^a^Regarding points assessed for basic autonomic function tests: see the Methods section.

To validate the implementation, we scored the SUs of the above mentioned items and created a Syncope Unit-19 score named as: SU-19 score. The SUs could score 15 points for adherence to consensus statement one (3 points for the structure and 12 points for the available diagnostic tests) and four points for adherence to consensus statement three. Both consensus statements^2^ were combined with the recommendations of the ESC guidelines on syncope.^1^

Regarding structure, the SUs could score a point on multidisciplinarity, integration within hospital organization/equipped facility, presence of internal protocols for diagnostic tests, and one point for every available diagnostic tests (12 tests). Regarding ‘basic autonomic function tests’ as available diagnostic test, these were not specified in the EHRA consensus statement one,^2^ therefore we followed the recommendation of the ESC guidelines on syncope.^1^ Basic autonomic function tests consist of: Valsalva manoeuvre, deep breathing 30:15 ratio, the cold pressure, sustained hand grip, and mental arithmetic. If one or more of these tests were available during the syncope evaluation, we rated the SU as meeting the requirement of the ‘basic autonomic function test’ and the SU scored one point.

Additionally, we tested the initial syncope evaluation as stated in the consensus statement three^2^ combined with the recommendations of the ESC guidelines^1^ on four points: history taking, physical examination, 12-lead ECG during initial evaluation, and an AST after 3 min.

Syncope units that comply fully to all requirements could score a maximum SU-19 score of 19 points.

All questionnaires were returned electronically in CastorEDC (https://www.castoredc.com). Whenever a questionnaire was not completed or returned, the investigator contacted the unit multiple times by phone or email. By filling out the questionnaire, the specialized SUs approved to participate in this survey. Syncope units were allowed to opt out at any time.

The ESC syncope guidelines prescribe a protocol for AST measurement as follows: first, a blood pressure measurement after 5 min of supine rest. The patient is then asked to stand up from supine, and blood pressure is measured after 3 min standing.^1,24^ We compared the guidelines recommendations to the protocol employed by the SUs. We also checked for AST measurements during sitting,^25^ after standing for 1 min,^24,26^ and 5 min.^27^ Syncope units that used a continuous blood pressure (CBP) monitor were excluded from this analysis as a CBP monitor takes measurements multiple times a second.^28,29^

The protocol of carotid sinus massage (CSM) per SU was compared to the ‘method of symptoms’.^1,30^ We compared the protocols of head-up tilt tests (HUTs) with the most feasible traditional ‘Italian’ protocol or FAST HUT protocol.^31,32^

We categorized the number of HUTs performed by a SU in groups of one to two, three to five, six to eight, and 9 to 15 HUTs per week (case load).

### Outcomes

The primary outcomes were: identification of all SUs in the Netherlands, specialties that are involved during assessment of the syncope patient, diagnostic tests available for syncope assessment, and to what extent SUs refer patients to academic SUs. Best practice as proposed by the EHRA consensus statements^2^ and ESC guidelines^1^ was evaluated per SU using the SU-19 score. Secondary outcomes were the case load per week and the number of autonomic function tests per evaluation.

### Adherence areas of the syncope units

The hospital adherence areas, defined as the regions and municipalities from which patients were referred to the SU, were obtained from the annual report from all hospitals that were included in this study. If these were not specified in the annual report, the communications department of the hospital was contacted. We obtained a comprehensive list of all municipalities in the Netherlands and obtained the adherence areas per municipality. We calculated the proportion of the total municipalities covered by the hospitals included in this study.

### Statistical analysis

Descriptive data were summarized using mean and standard deviation in case of a normal distribution and in case of non-normal distribution with median and interquartile range (IQR). Normality was tested by visual inspection of a Q-Q plot and was checked using skewness and kurtosis. The *z*-score was measured because sample size < 50. If the *z*-score was between −1.96 and +1.96 skewness and kurtosis, we assumed that there was a normal distribution.^33^ Tests for significance between the SU-19 score for non-academic and academic SUs were conducted using an independent *t*-test with a 95% confidence interval. A *P*-value of <0.05 was considered significant.

## Results

### Identification of syncope units and adherence areas

**Figure 1:**
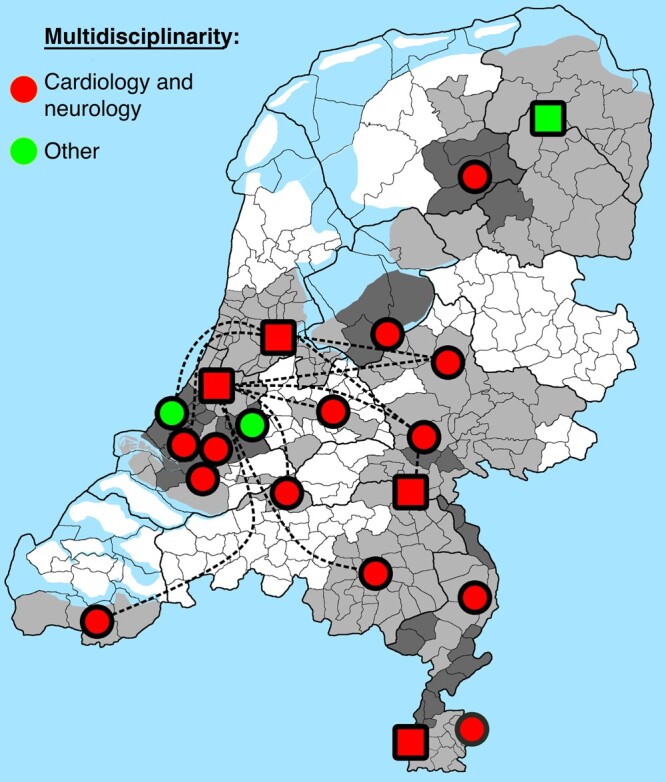
National overview of all academic and non-academic syncope units, their specialties involved, and their adherence areas in the Netherlands. Square blocks (□) indicate academic syncope units and circles (◯) indicate non-academic syncope units. Light grey areas indicate the adherence areas of syncope units and dark grey indicates overlap in adherence. White areas indicate that there is no formal adherence with a syncope unit. Dashed line indicates referral pattern to academic syncope unit. The image of the Netherlands is obtained from Wikipedia and the content of the municipalities has been removed and filled with light- and dark grey.

All 72 hospitals in the Netherlands were identified by ‘the national institute for public health’^23^ with in total 216 outpatient clinics cardiology, neurology, and internal medicine. All 216 outpatient clinics (72 × 3) neurology, cardiology, and internal medicine were contacted by phone. In 28% (20/72) of the hospitals, at least one outpatient clinic reported the presence of specialized care for syncope patients. All 20 clinics were included in this survey and are referred as SUs. All 20 SUs filled out the questionnaire. Our findings were cross-checked by the Dutch Autonomic Society, which did not yield any additional SUs. This makes the response rate 100% for this survey. For an overview of locations of the SUs and their respective adherence areas, see *Figure 1*. Seventy-five per cent of the SUs were located in non-academic hospitals (15/20). Eighty per cent (12/15) of the non-academic SUs report referral of patients to academic SUs. The remaining 20% (3/15) of non-academic SUs did not report referring patients. The reasons for referral were unexplained T-LOC (4/12), second opinion (3/12), confirmation of suspected epileptic seizures (2/12), psychogenic non-epileptic attacks (1/12), psychogenic pseudosyncope (1/12), or additional analysis for autonomic failure (1/12).

One hundred and twenty-two of the municipalities in the Netherlands (35.7%) were not covered by the adherence area of at least one of the included SUs. Forty-three (12.6%) municipalities were located within the adherence area of more than one SU (*Figure 1*). The case load of SUs was one to five patients per week in 60% (12/20), 5 to 10 patients per week in 35% (7/20), and more than 15 patients per week in 5% (1/20).

### Initial syncope evaluation

**Figure 2:**
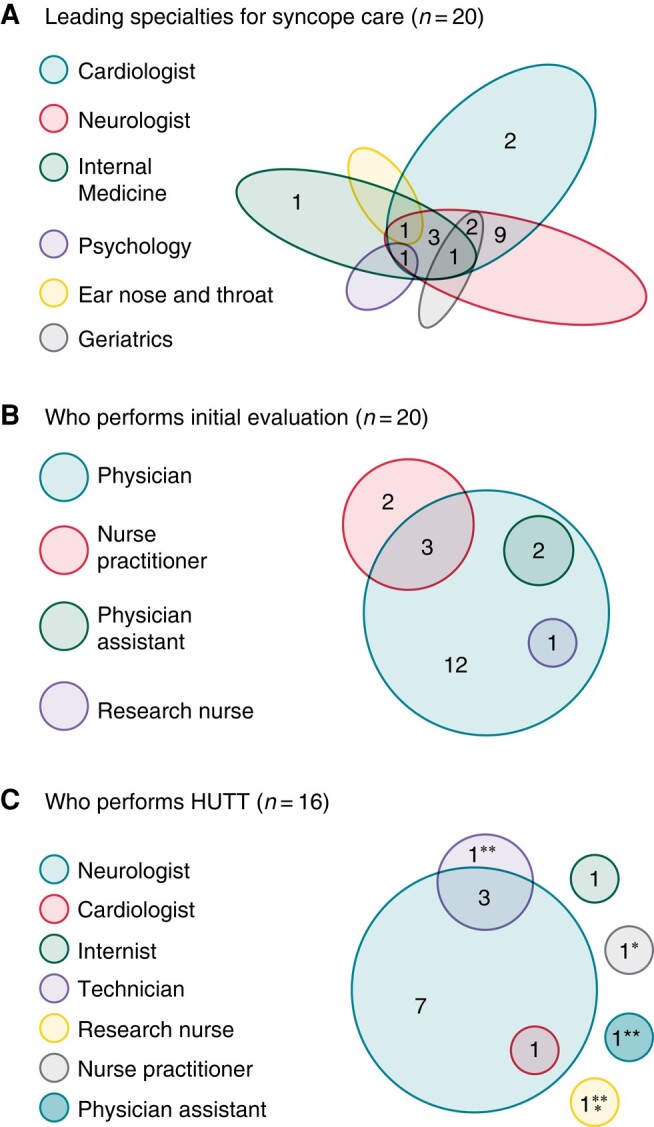
(*A*) Leading specialties for syncope care, numbers within the oval shapes indicate the specialties involved and their relative overlap, (*B*) who perform the initial evaluation and (*C*) head-up tilt tests. *Supervised by internist, **supervised by neurologist, ***supervised by cardiologist. HUT, head-up tilt test.

Multidisciplinarity was reported in 85% (17/20) of the SUs, all 17/20 involved cardiology and neurology for the initial syncope evaluation. Besides cardiology and neurology, also internal medicine (6/17), geriatric (3/17), ear nose and throat specialist (1/17), and psychology (1/17) were involved. In 10% (2/20), the initial evaluation was carried out exclusively by the cardiology department and in 5% (1/20) by the internal medicine department alone. All of them had appropriate access for multidisciplinary consultation when necessary (*Figure 2A*).

The initial evaluation was performed by a physician in 60% (12/20), or nurse practitioner 15% (3/20), physician assistant 10% (2/20), or research nurse 5% (1/20) under direct supervision of a physician (*Figure 2B*). In 10% (2/20) of the Sus, the nurse practitioner performed the initial evaluation alone and discussed the new cases on a multidisciplinary meeting (*Figure 2B*).

**Figure 3:**
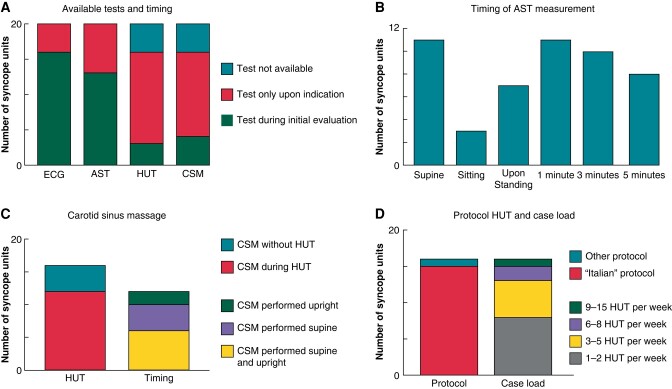
Multiple bar chart, showing an overview of the differences between syncope units regarding (*A*) available tests and timing, (*B*) active standing test, (*C*) carotid sinus massage, and (*D*) head-up tilt test. ECG, electrocardiogram; AST, active standing test; HUT, head-up tilt test; CSM, carotid sinus massage.

All SUs performed thorough history taking and physical examination. A 12-lead ECG was acquired during the initial evaluation in 80% (16/20) and during further assessment after initial evaluation in the other 20% (4/20) (*Figure 3A*).

#### Active standing test

Sixty-five per cent (13/20) of the SUs performed AST within their initial evaluation. The other 35% (7/20) performed this upon indication (*Figure 3A*). A CBP monitor was present in 80% (16/20) of the SUs. A CBP monitor was employed during AST, without use of a HUT in 45% (9/20), of which 33% (3/9) did not measure in sitting position and let the patient stand immediately. Twenty-two per cent (2/9) did not measure after 5 min of active standing but stopped AST after 3 min.

Fifty-five per cent (11/20) of the SUs performed AST with an upper-arm cuff, all performed the first measurement in supine position (11/11), followed during sitting in 27% (3/11), immediately after standing up in 64% (7/11), after standing for 1 min in all SUs (11/11), after standing for 3 min in 91% (10/11), and after standing for 5 min in 73% (8/11).

Active standing test was performed according to the ESC guidelines^1^ in 95% (19/20), only one (1/20) SU lacked a measurement after 3 min of standing, but did perform an AST measurement after 5 min (*Figure 3B*).

#### Carotid sinus massage

Eighty per cent (16/20) of the SUs performed CSM during syncope assessment (*Figure 3C*). Twenty-five per cent (4/16) did CSM during the initial evaluation, the other 75% (12/16) upon indication during additional syncope assessment (*Figure 3A*). Seventy-five per cent (12/16) performed CSM during the HUT; thirty-three per cent (4/12) only in supine position, 17% (2/12) only in head-up tilt position, and 50% (6/12) in both supine and upright position (*Figure 3C*). The four SUs that performed CSM without HUT did this without CBP monitor.

#### Head-up tilt test

A HUT could be performed in 80% (16/20) of the SUs of which 19% (3/16) performed the HUT during the initial evaluation (*Figure 3A*). All but one SU performed a HUT according to the ‘Italian’ protocol^31^ (*Figure 3D*). All SUs performed a HUT with a CBP monitor. Thirteen per cent (2/16) of SUs performed a hyperventilation test besides HUT.

The HUT was either performed or supervised by a neurologist in 75% (12/16), an internist in 13% (2/16), a cardiologist in 6% (1/16), and both a cardiologist and neurologist in 6% (1/16) (*Figure 2C*). Non-physicians were involved performing HUTs in 44% (7/16). Head-up tilt tests were performed alone by nurse practitioner in 6% (1/16), a physician assistant in 6% (1/16), a technician in 6% (1/16), or research nurse in 6% (1/16) (*Figure 2C*). The 16 clinics that used HUT performed 1 to 15 HUTs per week (median three HUTs per week; IQR: 2–4) (*Figure 3D*).

### The SU-19 score for syncope units

**Figure 4:**
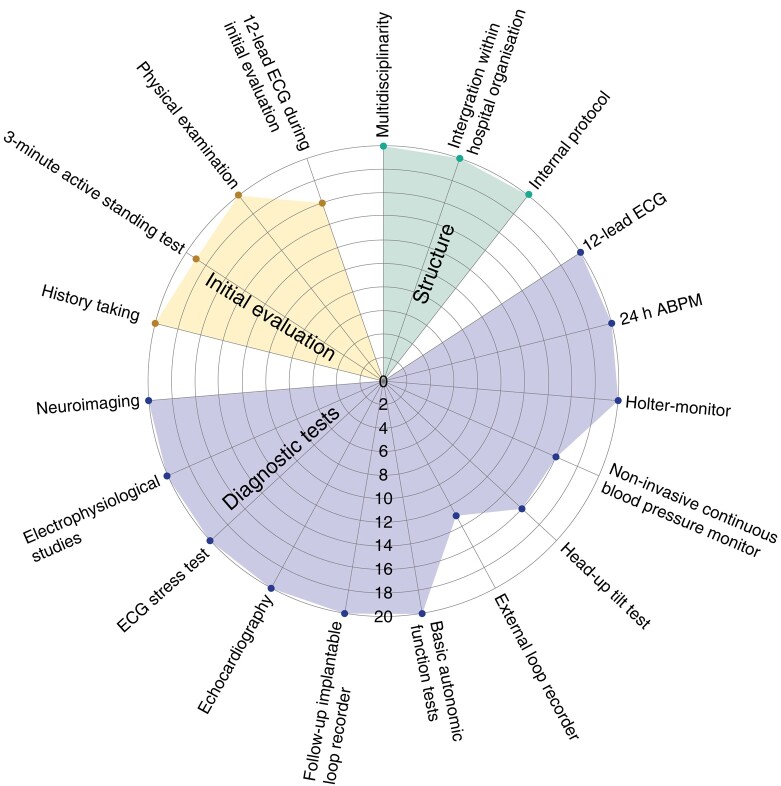
Responses shown in a spider graph for all 20 syncope units based on the syncope unit-19 score for best practices on structure (maximum of three points), diagnostic tests (maximum of 12 points), and initial evaluation (maximum of 4 points) based on European Heart Rhythm Association consensus statements one and three and the recommendations of the 2018 European Society of Cardiology guidelines on syncope. *The score on basic autonomic functions tests is specified in the Methods section. ECG, electrocardiogram; ABPM, ambulatory blood pressure monitoring.

The mean of the SU-19 score was 18.0 ± 1.1 (range: 16 to 19). All requirements of the SU-19 score were met in 45% (9/20), see *Figure 4*. All SUs complied to the EHRA consensus statement one^2^ on the following components: multidisciplinary, integration within hospital organization/equipped facility, presence of internal protocol, possibility of a 12-lead ECG, Holter monitor, follow-up of implantable loop recorders, 24 h ambulatory blood pressure monitoring (ABPM), basic autonomic function tests, access to echocardiography, electrophysiological studies, and ECG stress test and neuroimaging (*Figure 4*).

The mean of the SU-19 score for the academic hospitals was 18.6 ± 0.9 (range: 17 to 19); for the non-academic, this was 17.8 ± 1.1 (range: 16 to 19) (*P* = 0.174).

The initial evaluation was done in compliance with the ESC guidelines^1^ and EHRA consensus statement three^2^ in all SUs with regard to history taking and physical examination (*Figure 4*).

## Discussion

### The structural approach of the Dutch syncope units

All 20 SUs we identified (5 academic, 15 non-academic) implemented sufficient amount of time for thorough history taking, conducted a complete physical examination including AST and 12-lead ECG. Implementation of guidelines remains challenging in the emergency department.^8^ Our national survey shows that implementation of guidelines based syncope care in the SUs appears to be much better than expected.

Contrary to our primary hypothesis, SUs generally shared a similar approach, with notable differences in organizational and diagnostic approach. Only four SUs did not utilize a 12-lead ECG during the initial evaluation. Most SUs followed a consistent protocol for AST, but one SU recorded a second blood pressure after five instead of 3 min of standing, but opted for measurement after 5 min. A simple adjustment in the protocol can align all SUs for best practices for 12-lead ECG and AST.^2^ The definition of orthostatic hypotension requires a systolic blood pressure fall of over 20 mL of mercury and/or a diastolic blood pressure fall of over 10 mL of mercury after 3 min standing, precluding a definitive diagnosis when a 3 min measurement is not taken.^1,24^ Detecting initial orthostatic hypotension without a CBP monitor is challenging due to decrease in blood pressure that may have passed before the cuff is able to deflate completely.^24,34,35^ Therefore, a CBP monitor is crucial for diagnosing initial orthostatic hypotension,^34^ especially upon sitting and immediately after standing up.

Carotid sinus massage was performed in almost all of the SUs (*Figure 3C*) although without the use of a CBP monitor in four SUs. Again, beat-to-beat CBP is needed to relate the instant blood pressure fall.^1,9^ Carotid sinus massage is associated with a slight risk of a transient ischaemic attack or stroke^30,36–39^ and should not be performed unless adequate, guidelines recommended measurements can be obtained during the manoeuvre. Carotid sinus massage should be performed in the supine position on both carotid arteries and when non-diagnostic, in head-up tilt position.^1,9,40–44^ We realize that high costs hamper the use of CBP for CSM and AST measurement.^8^ Regarding the protocol used for CSM, there is variation in the position of the patients during CSM and simple adjustments in the protocol results in best practice.^42^

The majority of SUs utilized HUT tests to evaluate patients with T-LOC; predominantly located in neurology departments. Traditional Italian HUT protocols lasting at 45 min, may limit, and expertise is crucial for proper interpretation.^13,45^ However a fast Italian protocol maintains diagnostic accuracy while reducing testing time, as highlighted by Russo *et al*.^32^

This survey shows that in almost 50% of the HUTs were done by non-physicians and these were trained to perform HUTs alone (*Figure 2C*). This growing group of specialized non-physicians is becoming increasingly important in staffing the SU,^1^ perhaps this can help the four SUs that do not have HUT at their disposal in daily practice to have one and thereby improve syncope care in their clinic.

### SU-19 score for best practices: to compare implementation of guidelines on syncope in academic and non-academic syncope units

The SU-19 score provides a straightforward method to assess the adherence syncope guidelines in SUs (see *Figure 4*). Although the academic SUs scored slightly better than the non-academic, the SU-score does not differ significantly (*P* = 0.174). Notably, non-academic SUs demonstrated impressive implementation of guidelines based syncope evaluation. Most SUs met the requirements for structure of the consensus statements and the ESC guidelines (*Figure 4*, green).^1,2^ Potentially, the absence of a CBP monitor, HUT, and external loop recorders in some SUs (*Figure 4*, purple) is probably caused by high costs.^8^ One may question whether outpatient clinics without a HUT should be called a SU. This issue cannot be solved with our uses of a questionnaire from self-reported SUs. External loop recorders are not widely available in the Netherlands, similar to most other countries.

The initial evaluation (*Figure 4*, yellow), according to ESC guidelines^1^ and consensus statement three,^2^ was adequately implemented with minor discrepancies identified, particularly in performing AST measurement at 3 min after standing and a 12-lead ECG. One could hypothesize that these core assessments should be performed directly upon the initial syncope evaluation, and should not be too difficult. However, organizational context of the hospital may however hamper this.^8^

After completing our survey, the study on 24 h ABPM in patients with reflex syncope was published,^46,47^ strengthening the indication for this diagnostic test in daily practice and should be present in every SU.

These results show that minor adjustments of the local protocols or organizational context will make all the included SUs in the Netherlands meet the requirements for EHRA consensus statements one and three^2^ and the recommendations of the ESC guidelines on syncope^1^ for best practice and score maximal for the SU-19 score.

As highlighted in the introduction, comprehensive management of syncope patients involves not only diagnostics but also treatment considerations. The SU-19 score currently addresses only consensus statements one (structure and the availability of diagnostic tests) and three (initial evaluation), without incorporating the evaluation of treatment of the syncope patient. This survey did not access the aspect of treatment and should be added to more comprehensive assessment of SUs. Further investigation is warranted to delineate this aspect, necessitating an adaptation of the SU-19 score to align with the implementation of guideline based care regarding diagnostic evaluation and treatment of the SU.

### Which specialties are active in syncope care?

The SUs were managed by more than one medical specialty (*Figure 1*), or appropriate access for multidisciplinary consultation was guaranteed. This demonstrates that syncope is considered a condition that reaches beyond a single medical specialty.^1,2,8^ Notably, cardiologist and neurologists were the most frequently involved specialties, with neurologists primarily performing HUT. Indeed, the most threatening causes of syncope, i.e. cardiac arrhythmias, structural heart diseases, and epilepsy, are covered by cardiologists and neurologist, but other causes of T-LOC are more prevalent than the cardiac and neurological causes.^5^ Specialists often focus on their specific expertise, potentially leading to neglect of syncope diagnoses outside their domain.^48,49^ Therefore, cardiologist and neurologist should take the lead in addressing non-cardiac causes of syncope.^49–51^

Setting up SUs remains a challenge, not only in the Netherlands but also in other countries and parts of the world.^6^ Contemporary outpatient consultation is confronted with limited time, making it difficult to take a thorough history, and virtually impossible to build a history.^52^ Nevertheless, SUs could provide a protocol based evaluation that is best suitable for their own healthcare system within their own unit^6^ following guidelines recommendation.

### National network of syncope units

There is an incentive for establishment of SUs with a structured or standardized approach, as this improves diagnostic yield, and lowers the healthcare costs by reducing hospital admission.^4–7^ This study shows that both academic and non-academic hospitals are capable of implementing guidelines based syncope care. As patients with syncope require a multidisciplinary approach, a structured diagnostic strategy including rational use of diagnostic test may avoid overuse of resources.^5,6,51^ With the support of national working groups (e.g. The Dutch Autonomic Society) or (online) platforms such as Syncopedia,^53^ this survey may help to initiate writing a handbook, any form of certification/accreditation to assist existing SUs with improving their care. The justification for the establishment of SUs lies in the ability to establish, confirm, and/or explain the diagnoses to the patient. This concerns mainly the non-cardiac diagnoses of syncope that are frequently missed.^5,49,51^

In 2019, ∼2 million (2 039 000) emergency department presentations were registered in the Netherlands,^54^ 1–3% of emergency department presentations were due to T-LOC estimating 20 390 to 61 170 patients annually.^49,55–57^ Current SUs handle a maximum of 150 patients weekly, potentially leaving 1026 patients without SU referral. Considering that 11 600 hospitalizations in 2019^54^ and 150 weekly, 803 patients weekly were not referred. Vasovagal reflex syncope is the leading cause (78–85% accuracy), followed by orthostatic hypotension (57–100% accuracy).^58,59^ Emergency department diagnoses of these conditions range from 67.4% to 81.2%,^58,59^ suggesting that 151 patients weekly should be referred to an SU, potentially not in every hospital (*Figure 1*).

This study is a nationwide study and therefore conclusion regarding other countries should be made with caution. As the Netherlands appears to have the highest concentration of autonomic nervous system laboratories in Europe,^60^ the inability of hospitals to refer patients for syncope care is probably limited throughout Europe.

### Limitations of this study

This study was limited by its cross-sectional design with the use of a questionnaire. Every effort was made to approach all hospitals and all neurology, cardiology, and internal medicine units separately, but there is a possibility that some hospitals having a form of syncope care were missed, newly started, or stopped. However, after identification of the Sus, the Dutch Autonomic Society was consulted to confirm the identified SUs and none were missed according to their knowledge.

The survey interrogation of SUs did not include the exact content of history taking (which questions were asked) and physical examination (regardless the detailed AST). This might have influenced the outcome and we will include this in further upcoming analyses. The setup was purely qualitative and not quantitative.

Although this study was conducted only in the Netherlands, it is interesting to consider this in a broader perspective, such as in Europe. Using the SU-19 score provides an easy and cheap method to assess quickly for outpatient clinics in Europe, or by country, to see how they comply with both the EHRA consensus statement and ESC guidelines on syncope.^1,2^ Additionally, it can serve as an effective means, with the differences in medical healthcare systems, to demonstrate the healthcare insurance companies what is required and what improvements may be needed.

The formal adherence areas of the hospitals are shown, but we did not investigate how units in areas without a SU refer patients. As a result, the adherence area could be larger than is currently shown since the obtained data do not allow analysis of whether patients with syncope within an adherence area were in fact referred to the appropriate SU in that area.

## Conclusion

We identified 20 SUs in the Netherlands, 5 were located in academic and 15 were located in non-academic hospitals. Cardiologists and neurologists are mainly involved in the management of syncope patients, but the role of non-physicians is growing. All SUs had a high SU-19 score and broadly implemented the EHRA consensus statements one and three combined with the recommendations of the ESC guidelines on syncope.^1,2^ The current heterogeneity between SUs can be minimized by an operational agreement on syncope care with clear criteria on initial evaluation, structure, and diagnostic tests available in the SU.

## Supplementary Material

euae002_Supplementary_Data

## Data Availability

All relevant data are within the manuscript and its supporting information files.
