# Supplementary material for: A cross-sectional nationwide survey of guideline based syncope units in the Netherlands: the SU-19 score—a novel validation for best practices
Source: Europace. 2024 Jan 8;26(1):euae002. doi: 10.1093/europace/euae002 (PMC10793571; doi:10.1093/europace/euae002)
Supplement: euae002_Supplementary_Data [file euae002_supplementary_data.docx]

# Supplementary material

**Supplementary 1**

**Survey “Questionnaire Syncope Network second and third line care in the Netherlands”**

**1. Syncope Unit**

1.1 Is there a Syncope Unit specifically for syncope patients within your organization?

○ Yes

○ No (proceed to question 2.1)

1.2 How many patients with syncope are seen weekly at the Syncope Unit?

○ 0

○ 1 to 5

○ 5 to 10

○ 10 to 15

○ 15 to 20

○ 20 to 25

○ > 25

1.3 Who performs the evaluation of syncope patients at the Syncope Unit? (multiple answers possible)

○ Physician

○ Nurse Practitioner

○ Physician Assistant

○ Other, ………………………

1.4 Which diagnostics are performed within the initial evaluation? (multiple answers possible)

○ History taking

○ Thorough history taking

○ Physical examination (general)

○ Physical examination (only cardiology)

○ Physical examination (only neurology)

○ ECG (12-lead)

○ Blood pressure measurement

○ Carotid Sinus massage

○ Orthostatic blood pressure measurement

○ Echocardiogram

○ ECG stress test

○ Holter monitoring

○ Head-up tilt test

○ CT-cerebrum

○ MRI-cerebrum

○ Epley manoeuvre

○ Other, ………………………

1.5 Which diagnostics are performed within the additional evaluation? (multiple answers possible)

○ None

○ History taking

○ Thorough history taking

○ Physical examination (general)

○ Physical examination (only cardiology)

○ Physical examination (only neurology)

○ ECG (12-lead)

○ Blood pressure measurement

○ Carotid Sinus massage

○ Orthostatic blood pressure measurement

○ Echocardiogram

○ ECG stress test

○ Holter monitoring

○ Head-up tilt-test

○ CT-cerebrum

○ MRI-cerebrum

○ Epley manoeuvre

○ Other, ………………………

1.6 Is multidisciplinary part of the Syncope Unit?

○ Yes

○ No (proceed to question 2.1)

1.7 If yes, which discipline? (multiple answers possible)

○ Cardiologist

○ Neurologist

○ Internist

○ Ear, nose and throat specialist

○ Physiotherapist

○ Other, ………………………

**2. Head-up tilt test**

2.1 Is there a head-up tilt test in the hospital?

○ Yes

○ No (proceed to question 3.1)

2.2 What is the question for head-up tilt test?

……………………………………………..

2.3 What is the reason for head-up tilt test and which protocol is used?

……………………………………………..

2.4 Is active standing part of the head-up tilt test?

○ Yes

○ No

○ I don’t know

2.5 Is the head-up tilt test with supine carotid sinus massage?

○ Yes

○ No

○ I don’t know

2.6 Is the head-up tilt test with upright carotid sinus massage?

○ Yes

○ No

○ I don’t know

2.7 Nitroglycerine is used as part of the head-up tilt test?

○ Yes

○ No

○ Only on indication

○ I don’t know

2.8 Who performs the head-up tilt test? (multiple answers possible)

○ Cardiologist

○ Neurologist

○ Internist

○ Other, ………………………

2.9 How many head-up tilt tests are performed per week?

……………………………………………..

**3. Orthostatic blood pressure measurement**

3.1 Is orthostatic blood pressure measurement part of the initial evaluation?

○ Yes (proceed to question 3.3)

○ No

3.2 Why not?

○ Only on indication

○ Use of head-up tilt test

○ No added value

○ Other, ………………………

3.3 How is the orthostatic blood pressure measurement test performed? (multiple answers possible)

○ Supine

○ Sitting

○ Up on standing

○ After standing 1 minute

○ After standing 3 minutes

○ After standing 5 minutes

3.4 What equipment is used? (multiple answers possible)

○ Upper-arm cuff and stethoscope

○ Electronic blood pressure meter

○ Continuous blood pressure monitor

○ Other, ………………………

**4. Referral**

4.1 Are syncope patients referred from your unit to a tertiary Syncope Unit?

○ Yes

○ No (proceed to question 5.1)

4.2 To which hospital are syncope patients referred?

……………………………………………..

4.3 What is the referral question?

……………………………………………..

4.4 How many syncope patients are referred per year?

……………………………………………..

**5. Additional comments**

5.1 Are there any other comments or additional information you would like to add?

………………………………………………………………………………………………………………………………………………………………………………………………………………………………………………………………………………………………………………………………………………………………………………………………………………………………………………………………………………………………………………………………………………………………………………………………………………………
